# Diagnostic and Prognostic Value of Cardiac Magnetic Resonance for Cardiotoxicity Caused by Immune Checkpoint Inhibitors: A Systematic Review and Meta-Analysis

**DOI:** 10.31083/RCM25508

**Published:** 2025-02-21

**Authors:** Jialian Li, Yanwei Li, Li Tao, Chuan Zhang, Zhong Zuo

**Affiliations:** ^1^Department of Cardiology, The First Affiliated Hospital of Chongqing Medical University, 400016 Chongqing, China; ^2^Department of Radiology, The First Affiliated Hospital of Chongqing Medical University, 400016 Chongqing, China

**Keywords:** cardiac magnetic resonance, immune checkpoint inhibitors, cardiotoxicity, global longitudinal strain, global radial strain, T1, T2

## Abstract

**Background:**

The complex process of cardiac magnetic resonance (CMR) and the uncertainty of each parameter in the diagnosis and prognosis of cardiotoxicity limit its promotion in the cardiac evaluation of patients treated with immune checkpoint inhibitors (ICI).

**Methods:**

A comprehensive search was conducted across PubMed, Web of Science, Embase, China National Knowledge Infrastructure (CNKI), and Cochrane databases for relevant articles published up until September 28, 2024.

**Results:**

After screening, 8 articles were included in this study. The analysis revealed that following ICI treatment, the left ventricular global longitudinal strain (GLS) increased significantly [weighted mean difference (WMD) 2.33; 95% confidence interval (CI) 1.26, 3.41; *p* < 0.01], while the global radial strain (GRS) decreased [WMD –4.73; 95% CI –6.74, –2.71; *p* < 0.01]. Additionally, T1 and T2 values increased [standardized mean difference (SMD) 1.14; 95% CI 0.59, 1.68; *p* < 0.01] and [SMD 1.11; 95% CI 0.64, 1.58; *p* < 0.01], respectively. An elevated T2 was associated with a higher occurrence of major adverse cardiovascular events (MACE), with a hazard ratio of 1.36 (95% CI 1.12, 1.64).

**Conclusions:**

Our findings demonstrate that T1, T2, and GLS increase, while GRS decreases following ICI administration. By consolidating these critical metrics, we propose a streamlined, abbreviated (non-contrast) CMR protocol that can be completed within 15 minutes, thereby facilitating the integration of CMR in cardio-oncology.

**The PROSPERO registration:**

CRD42023437238, https://www.crd.york.ac.uk/prospero/display_record.php?ID=CRD42023437238.

## 1. Introduction

Tumor cells can evade the immune system through mechanisms such as molecular mimicry, which includes immune checkpoint proteins (IC) [1]. Immune checkpoint inhibitors (ICI) work by blocking this pathway to elicit anti-tumor effects [2]. However, in the process of inhibiting the IC of tumor cells, these therapies may also interfere with the IC of normal organs and tissues, leading to immune-related adverse events (irAEs). Following the FDA’s (Food and Drug Administration) approval of ipilimumab for the treatment of advanced melanoma [3], the utilization of ICI across various malignancies has become increasingly prevalent. Additionally, there has been a growing number of reports of irAEs [4], including ICI-related myocarditis (ICI-M) [5] and other cardiovascular related irAEs (non-inflammatory forms of heart failure) [6] are of concern because of their high mortality rates [5]. Early systematic screening may help reduce these cardiotoxicities [7]. Cardiac magnetic resonance (CMR) may be used in the early identification of cardiotoxicity due to its unique advantages.

CMR exhibits robust tissue characterization capabilities, enabling non-invasive assessment of myocardial lesions [8]. It facilitates the detection of early myocardial damage, such as inflammation and edema, while also allowing for distinctive identification of myocardial fibrosis. Moreover, CMR demonstrates excellent measurement reproducibility, offering precise quantitative assessment information that is unattainable through alternative imaging examinations [8]. The 2022 European Society of Cardiology (ESC) cardio-oncology guidelines also emphasizes the important value of CMR [9]. However, the complex process of CMR hinders its dissemination for cardiac estimation in the hearts of ICI patients [10]. CMR is used as an alternative to transthoracic echocardiography (TTE) [11].

This meta-analysis aims to evaluate the diagnostic and prognostic significance of various CMR indicators in relation to ICI-induced cardiotoxicity. It seeks to propose a novel CMR protocol for monitoring ICI-related cardiotoxicity, with the goals of reducing the duration of CMR procedures, lowering costs, and enhancing the accessibility of CMR in the field of cardio-oncology, ultimately providing more valuable clinical information.

## 2. Materials and Methods

### 2.1 Search Strategy and Selection Criteria

This meta-analysis was conducted according to the Preferred Reporting Items for Systematic Reviews and Meta-Analysis (PRISMA) reporting guidelines [12]. The study was prospectively registered and accessed under PROSPERO (CRD42023437238). PubMed, Web of Science, Embase, China National Knowledge Infrastructure (CNKI), and Cochrane were screened for studies that assessed the value of CMR in cardiotoxicity related to ICI treatment. The search included papers published from database inception until September 28, 2024. The search strategy, including query terms used to identify relevant research articles, is summarized in Table 1 [13]. Two reviewers independently screened the literature. A third individual was consulted in any cases of disagreement.

**Table 1.** **Search terms for literature review strategy**.

Query category	Search terms
Immune-related adverse events (irAEs)	Immune-related adverse events (irAEs [TIAB])
Cardiac-related	- Cardiotoxicity: “Cardiotoxin” [MeSH], cardiotox* [TIAB]
	- Heart Failure: “Heart Failure” [MeSH], cardiac failure* [TIAB], myocardial failure*[TIAB]
	- Myocarditis: “myocarditis” [MeSH], myocarditis [TIAB]
	- Pericarditis: “pericarditis” [MeSH], pericarditis [TIAB], “pericardial effusion” [MeSH], (“pericardial” [TIAB] AND “effusion” [TIAB])
	- Heart Arrest: “Heart Arrest” [MeSH], “heart arrest” [TIAB], (“heart” [TIAB] AND “arrest” [TIAB]), (“cardiac” [TIAB] AND “arrest” [TIAB])
	- Acute Coronary Syndrome: “acute coronary syndrome” [MeSH]
	- Takotsubo-like syndrome: “Takotsubo-like syndrome” [TIAB]
	- Arrhythmias: “Arrhythmias, Cardiac” [MeSH], arrhythmias [TIAB], (“arrhythmias” [TIAB] AND “cardiac” [TIAB]), “cardiac arrhythmias” [TIAB], fibrillation [TIAB]
	- Vasculitis: “vasculitis” [MeSH] OR vasculitis [TIAB]
	- Myocardial Infarction: “myocardial infarction” [MeSH], (“myocardial” [TIAB] AND “infarction” [TIAB])
Magnetic resonance imaging	“Magnetic Resonance Imaging” [MeSH], MR [TIAB], NMR* [TIAB], MRI [TIAB], Chemical Shift Imaging* [TIAB], CMR [TIAB]
Checkpoint inhibitors	- CTLA-4 Inhibitors: “ctla-4 antigen” [MeSH], CTLA-4* [TIAB], CTLA-4 inhibitor* [TIAB], ipilimumab* [TIAB]
	- PD-1 Inhibitors: PD-1* [TIAB], PD-1 inhibitor* [TIAB], nivolumab* [TIAB], pembrolizumab* [TIAB]
	- PD-L1: PD-L1* [TIAB], PD-L1 inhibitors [TIAB], atezolizumab* [TIAB], avelumab* [TIAB], tremelimumab* [TIAB], cemiplimab* [TIAB], durvalumab* [TIAB], dostarlimab* [TIAB]
	- Immune Checkpoint Inhibitors: “cell cycle checkpoints”[MeSH], Immune Checkpoint Inhibitors [TIAB], checkpoint inhibitor*[TIAB], check-point inhibitor*[TIAB], Immune Checkpoint Block*[TIAB]

TIAB, title/abstract; MeSH, Medical Subject Headings, is the National Library of Medicine controlled vocabulary thesaurus used for indexing articles for PubMed; MR, magnetic resonance; NMR, nuclear magnetic resonance; MRI, magnetic resonance imaging; CMR, cardiac magnetic resonance; CTLA-4, cytotoxic T-lymphocyte antigen 4; PD-1, programmed cell death protein 1; PD-L1, programmed cell death ligand 1. The symbol “*” is used as a wildcard in PubMed to perform truncated searches, allowing for the inclusion of multiple word variations.

### 2.2 Study Selection

The incorporation of standards was outlined as follows: (1) Patients undergoing treatment with ICI, (2) employing CMR for patient monitoring, (3) scrutinizing for cardiotoxicities such as congestive heart failure or myocarditis, (4) inclusion of randomized controlled trials, cohort studies, and case-control studies.

**Fig. 1.**
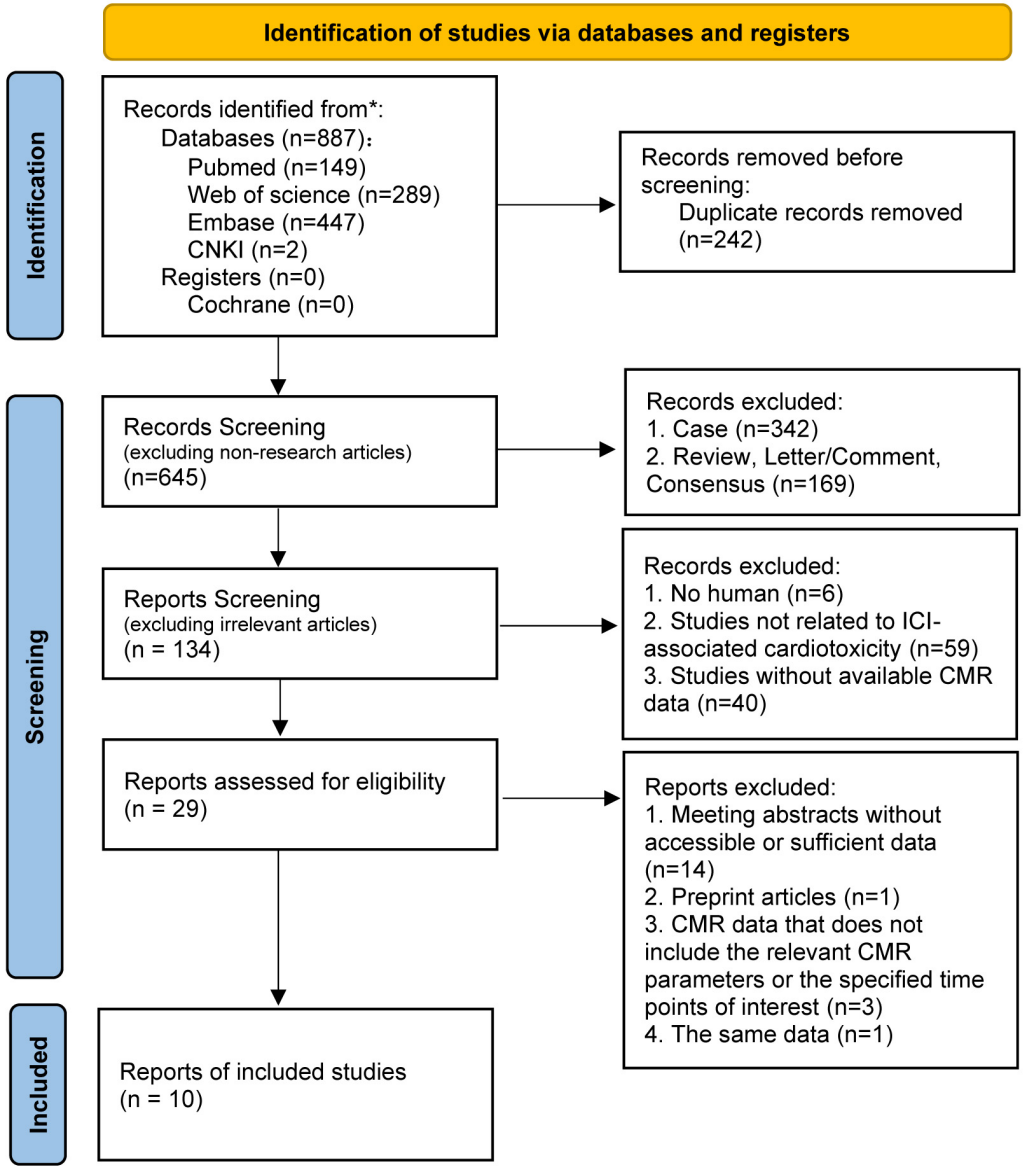
**PRISMA flow diagram**. CNKI, China National Knowledge Infrastructure, a full-text academic resource database; CMR, cardiac magnetic resonance; ICI, immune checkpoint inhibitor.

Exclusion criteria encompassed: (1) Case reports, reviews, letters, and consensus papers; (2) Non-human subjects; (3) Studies not related to ICI-associated cardiotoxicity; (4) Studies lacking available CMR data; (5) Meeting abstracts without accessible or sufficient data; (6) Preprint article; (7) CMR data not including relevant parameters or specified time points of interest; (8) Repeated data from the same study (Fig. 1).

### 2.3 Quality Assessment

The Newcastle–Ottawa Scale (NOS) for cohort studies was used to assess the quality of the included studies, and the assessment was performed by two independent reviewers (Table 2, Ref. [14, 15, 16, 17, 18, 19, 20, 21, 22, 23]). Studies were considered to be of high quality if they had an NOS score ≥6. Conversely, studies with a score <4 were deemed to be of low quality and subsequently excluded.

**Table 2.** **Quality assessment using the Newcastle–Ottawa Scale for cohort studies**.

Author	Selection				Comparability	Outcome			Total
	Representativeness of the exposed cohort	Selection of the non-exposed cohort	Ascertainment of exposure	Demonstration that outcome of interest was not present at start of study	Comparability of cohorts on the basis of the design or analysis controlled for confounders	Assessment of outcome	Was follow-up long enough for outcomes to occur	Adequacy of follow-up of cohorts	
Thavendiranathan *et al*. 2021 [14]	1	1	1	1	2	1	1	1	9
Zhang *et al*. 2020 [18]	1	1	1	1	2	1	1	1	9
Cadour *et al*. 2022 [19]	1		1	1	2	1	1	1	8
Zhao *et al*. 2022 [15]	1	1	1	1	2	1	1		8
Faron *et al*. 2021 [20]	1	1	1	1	1	1	1		7
Li *et al*. 2024 [21]	1	1	1	1	2	1			7
Liu *et al*. 2022 [22]	1	1	1	1	1	1	1		7
Mirza *et al*. 2022 [23]	1		1	1	2	1			6
Higgins *et al*. 2021 [16]	1		1			1			3
Tong *et al*. 2024 [17]	1		1			1			3

### 2.4 Data Extraction

The purpose of this study is to assess the occurrence of abnormal CMR indices following ICI therapy, whether they manifest or not, compared to baseline levels or reference values, and to determine their prognostic value. The primary outcome events consist of major adverse cardiovascular events (MACE), encompassing cardiovascular death, complete heart block, cardiogenic shock, and cardiac arrest [14].

Relevant outcomes involve examining the relationship between CMR biomarkers and the risk of cardiac toxicity and reporting hazard ratios (HRs). Data extraction was performed by two distinct researchers, both of whom evaluated any discrepancies. In cases where differences persisted, the final decision was made by a third author. Extracted data included publication year, study type, demographics, follow-up duration, outcome events, CMR types, CMR protocol, CMR parameter values, and outcome event-related results (Table 3, Ref. [14, 15, 18, 19, 20, 21, 22, 23]). Continuous variables extracted in this study are subsequently provided in the subsequent analyses. The variations in CMR parameter values, following the comparison of ICI-treated patients with their baseline values or local reference values as self-controls, are individually defined by each study.

**Table 3.** **Characteristics of studies included in the meta-analysis**.

Author	Sample size	Study type	Median follow-up (days)	Endpoints	Equipment	CMR sequence	CMR time^(1)^ (days)	Patient group	Control group
Zhao *et al*. [15] 2022, China	52	R	171	MACE	1.5T, Siemens	GLS, GCS, GRS; T1, T2 maps; LGE	7 (IQR: 3–10)	ICI-M	Healthy controls (n = 15)
Liu *et al*. [22] 2022, China	36	P	83	CTRCD	1.5T, Siemens	GLS, GCS, GRS; T1, T2 maps; ECV; LGE	Baseline, 3 weeks, 3 months	3 weeks, 3 months	Baseline (n = 36)
Mirza *et al*. [23] 2022, USA	8	R	-	-	1.5T, GM	GLS	-	ICI-M	Normal controls (n = 8)
Cadour *et al*. [19] 2022, France	33	R	92 + 33^(2)^	MACE	1.5T, 3.0T, Siemens; 1.5T, Philips	T1^(3)^, T2^(3)^ maps; ECV^(3)^; LGE	3 (IQR, 1–5) [after steroid introduction]	ICI-M	Pre-ICI group (n = 21)
Thavendiranathan *et al*. [14] 2021, USA	79	R	158	MACE	1.5T, 3.0T, Siemens; 1.5T, Philips	T1, T2 maps	58	ICI-M	Local reference value
Faron *et al*. [20] 2021, Germany	22	P	109	-	1.5T, Philips	GLS, GCS, GRS; T1, T2 maps; ECV	Baseline, 2 months	2 months	Baseline (n = 22)
Zhang *et al*. [18] 2020, USA	103	R	148.5	MACE	1.5T, 3.0T	LGE	LGE 6 (4–8); NO LGE 2 (1–5)^(4)^	ICI-M	-
Li *et al*. [21] 2024, China	35	P	32–70	-	1.5T, Siemens; 3.0T, Phillips	GLS; T1maps; ECV, LGE	2	ICI-M	Cancer patients

R, retrospective; P, prospective; CMR, cardiac magnetic resonance; MACE, major adverse cardiovascular events; CTRCD, cancer therapeutics-related cardiac dysfunction; GLS, global longitudinal strain; GRS, global radial strain; GCS, global circumferential strain; LGE, late gadolinium enhancement; ECV, extracellular volume; ICI-M, ICI-related myocarditis; ICI, immune checkpoint inhibitors; SD, standard deviation. ^(1)^ CMR time, time for performing CMR after hospital admission. ^(2)^ The median follow-up times for the ICI-M was 92 days (interquartile range [IQR], 16–317 days), and the MACE occurred after ICI-M with a median time of 33 days (IQR, 8–108 days). ^(3)^ The values of these sequences represent the Z-scores, which assess how many SDs each patient’s T1, T2, or ECV value deviates from the mean within the normal range for each site, vendor, and CMR field strength. ^(4)^ The time from admission to CMR was longer in patients with LGE (median time 6 days), compared to patients without LGE (median time 2 days, *p* < 0.001).

### 2.5 Statistical Analysis

In the incorporated studies, we extracted baseline and follow-up data, or patient observational values compared to reference values or control values, expressed as mean ± standard deviation (SD). We employed the inconsistency index (I^2^) and χ^2^-based Q test; I^2^ values of 25%, 50%, and 75% are considered low, moderate, and high estimates, respectively. Patients with I^2^ > 50% indicated significant heterogeneity [24]. In the presence of significant heterogeneity (I^2^ > 50%), statistical analysis was conducted using a random-effects model; otherwise (I^2^ ≤ 50%), a fixed-effects model was applied. For continuous variables, data were presented as the weighted mean difference (WMD). When the measurement units of the data were not constant, the standardized mean difference (SMD) was utilized. The inverse variance method was employed to calculate WMD or SMD and their corresponding 95% confidence intervals (CI). A generic inverse variance meta-analysis with a 95% CI was used to combine HRs. Data calculation was conducted using Review Manager (RevMan) version 5.4 (the Cochrane Collaboration, the Nordic Cochrane Centre, Copenhagen, Denmark).

## 3. Results

### 3.1 Selection Results and Characteristics

Through our search, we initially identified 887 articles. After removing 242 duplicate records and conducting a detailed screening process (Fig. 1), we ultimately included 10 studies [14, 15, 16, 17, 18, 19, 20, 21, 22, 23] in our analysis. Although Zhao authored two articles, we selected the one that provided more comprehensive data due to the significant overlap in their data collection periods [15], while excluding the other [25]. Additionally, three articles were excluded for not containing relevant CMR indicators of interest [26, 27] or for the timing of the provided CMR indicators not aligning with our requirements [28]. Following a literature quality assessment, we excluded two articles of lower quality [16, 17], resulting in the inclusion of CMR data from only 8 articles in the study. 6 studies [14, 15, 18, 19, 21, 23] assessed CMR in the diagnosis or clinical suspicion of ICI-M, while 2 [20, 22] focused on longitudinal follow-up using CMR for patients treated with ICI. 4 articles [15, 20, 22, 23] conducted assessments solely using 1.5T magnetic resonance imaging, while 4 [14, 18, 19, 21] utilized either 1.5T or 3.0T for evaluation. 4 studies [15, 19, 21, 23] involved comparisons with healthy individuals, pre-ICI control groups or cancer patients, 2 studies [20, 22] conducted comparisons with baseline data, 1 study [14] contrasted with local reference values, and no comparative information was found in 1 study [18]. 4 studies [14, 15, 18, 19] reported MACE events as the outcome, 1 study [22] focused on cancer therapeutics-related cardiac dysfunction (CTRCD), and information related to outcomes was not found in 3 studies [20, 21, 23]. Among these, 4 articles [15, 20, 21, 22] contain data concerning global longitudinal strain (GLS), while 3 articles [15, 20, 22] encompass information on both global radial strain (GRS) and global circumferential strain (GCS). However, none of them establish a relationship between CMR-feature tracking [CMR-FT (GLS, GRS and GCS)] and MACE. The collection comprises 5 articles [14, 15, 19, 20, 22] detailing native T1 and T2 values, with 3 of them [14, 15, 19] specifically addressing the association between native T1 and MACE, and 2 articles [14, 19] delving into the correlation between T2 and MACE. Furthermore, 2 articles [18, 19] focus on the relationship between late gadolinium enhancement (LGE) presence and MACE. Moreover, 4 articles [19, 20, 21, 22] include information on extracellular volume (ECV) values.

### 3.2 CMR Possesses the Capability to Diagnose ICI-Related Cardiotoxicity

The objective of the analysis is to evaluate specific parameters of CMR, such as CMR-FT (GLS, GRS, and GCS), and CMR tissue characterization, including T1 mapping, T2 mapping, ECV, and the presence of LGE, in patients undergoing ICI treatment and those experiencing cardiac toxicity such as myocarditis after ICI usage. This comparison was made against individuals not using ICI or normal reference values. The goal was to appraise the diagnostic efficacy of CMR.

#### 3.2.1 The CMR-FT

**Fig. 2.**
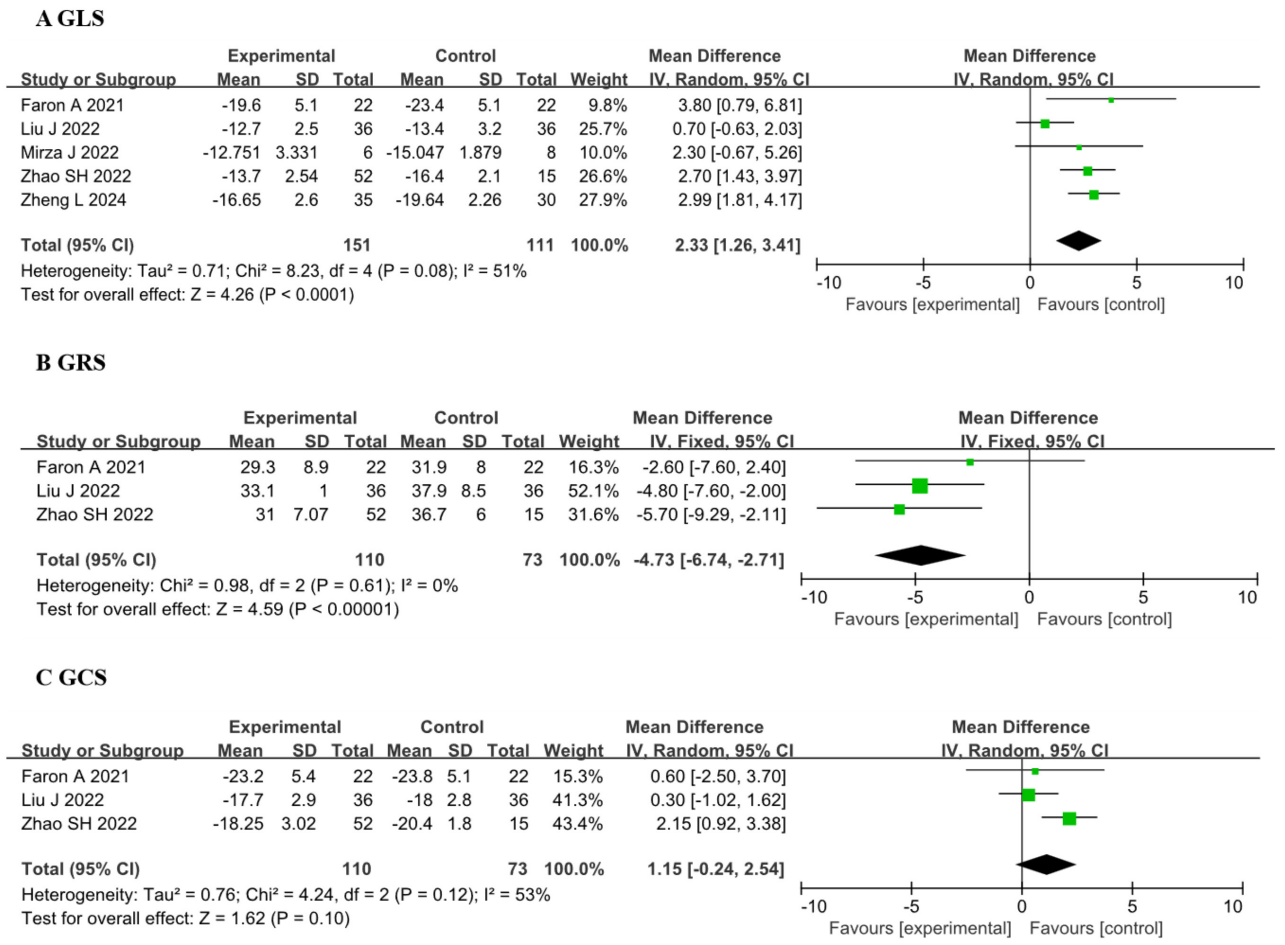
**Forest plots of the CMR-FT parameters before and after receiving ICI therapy**. (A) Comparison of GLS between before and after ICI therapy groups. (B) Comparison of GRS between before and after ICI therapy groups. (C) Comparison of GCS between before and after ICI therapy groups. Each plot shows the mean differences with 95% CI. GLS, global longitudinal strain; GRS, global radial strain; GCS, global circumferential strain; ICI, immune checkpoint inhibitors; CI, confidence interval; CMR-FT, cardiac magnetic resonance-feature tracking; IV, inverse variance.

Five studies [15, 21, 22, 23] involving a total of 151 patients demonstrated a significant difference in GLS after treatment compared to baseline or no treatment, with a WMD of 2.33 (95% CI 1.26, 3.41), *p* < 0.01. Additionally, three articles [15, 20, 22] involving 110 patients indicated that GRS was impaired, with a WMD of –4.73 (95% CI –6.74, –2.71), *p* < 0.01. Furthermore, there was no significant difference in GCS between the experimental group and the control group, with a WMD of 1.15 (95% CI –0.24, 2.54), *p* = 0.10 (Fig. 2).

#### 3.2.2 The CMR Tissue Characterization

**Fig. 3.**
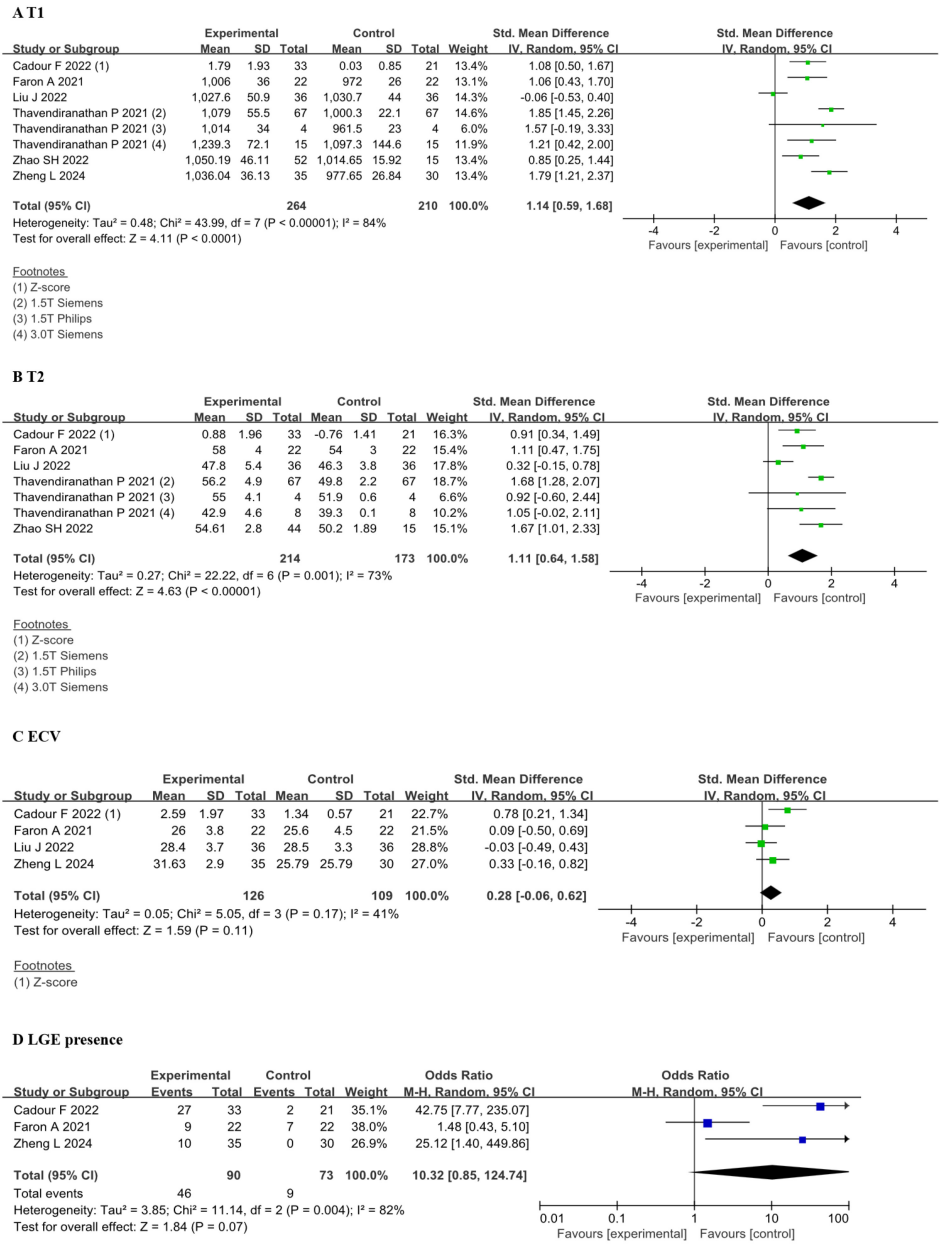
**Forest plots of the CMR tissue characterization parameters before and after receiving ICI therapy**. (A) Comparison of T1 value between before and after ICI therapy groups. (B) Comparison of T2 value between before and after ICI therapy groups. (C) Comparison of ECV between before and after ICI therapy groups. (D) Comparison of LGE presence between before and after ICI therapy groups. Each plot shows the mean differences or odds ratios with 95% CI. Z-score provides an assessment of how many SDs each patient’s T1, T2, or ECV value deviates from the mean within the normal range for each site, vendor, and CMR field strength. CMR, cardiac magnetic resonance; ICI, immune checkpoint inhibitors; CI, confidence interval; ECV, extracellular volume; LGE, late gadolinium enhancement; M-H, Mantel-Haenszel; IV, inverse variance.

The results indicated that the SMD for the T1 value was 1.14 (95% CI 0.59, 1.68, *p* < 0.01), while the SMD for the T2 value was 1.11 (95% CI 0.64, 1.58, *p* < 0.01), both of which were significantly elevated. In contrast, the SMD for the ECV was 0.28 (95% CI –0.06, 0.62, *p* = 0.11), indicating no significant change. Additionally, the odds ratio (OR) for LGE was 10.32 (95% CI 0.85, 124.74, *p* = 0.07), which did not achieve statistical significance (Fig. 3).

Significant heterogeneity was observed in the data, prompting us to conduct a reanalysis. We identified that the heterogeneity in T2 primarily originated from Liu J *et al*. [22]. Upon removal of this dataset, the results still demonstrated differences [SMD 1.33; 95% CI 1.02, 1.65; I^2^: 27%, *p* < 0.01]. Similarly, the heterogeneity in T1 was predominantly attributed to Liu J *et al*. [22] and Thavendiranathan *et al*. [14] (1.5T Siemens), and after excluding these datasets, the results continued to show differences [SMD 1.22; 95% CI 0.91, 1.52; I^2^: 14%, *p* < 0.01]. Due to the limited inclusion of studies and data, we were unable to analyze to determine the sources of heterogeneity in ECV.

Liu *et al*. [22] conducted a follow-up study utilizing data from 36 patients treated with ICI, wherein complications might not have been evident during the follow-up. Thavendiranathan *et al*. [14] performed evaluations on 67 ICI-M patients using a 1.5T Siemens machine, comparing the results with reference values.

Heterogeneity in this study is unavoidable. Regardless of the exclusion of certain values with significant differences, the results consistently demonstrate differences in T1 and T2 values, while ECV showed no significant disparity.

### 3.3 Prognosis

**Fig. 4.**
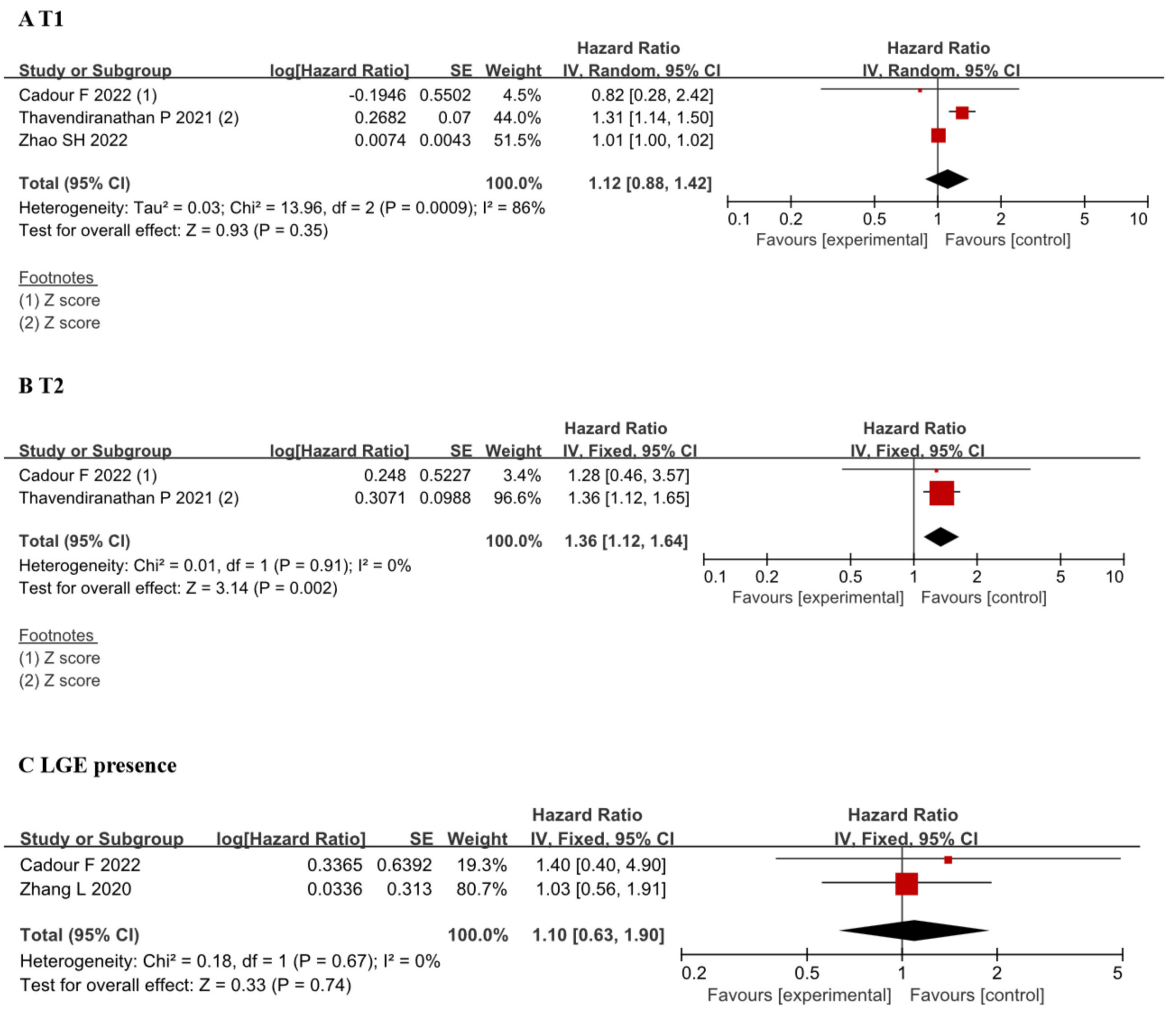
**Forest plots of the association between CMR tissue characterization (T1, T2, LGE presence) and MACE**. (A) Comparison of hazard ratios for T1 value between before and after ICI therapy groups. (B) Comparison of hazard ratios for T2 value between before and after ICI therapy groups. (C) Comparison of hazard ratios for LGE presence between before and after ICI therapy groups. Each plot shows the hazard ratios with 95% CI. ICI, immune checkpoint inhibitors; CI, confidence interval; LGE, late gadolinium enhancement; CMR, cardiac magnetic resonance; MACE, major adverse cardiovascular events; IV, inverse variance.

This study aimed to analyze the relationship between magnetic resonance parameters and prognosis. Our primary focus was on prognostic events, specifically MACE. Due to limitations in the data provided within the article, we exclusively analyzed the relationship between T1, T2, LGE, and the occurrence of MACE. We included three studies [14, 15, 19] to assess the relationship between T1 and MACE, yielding a HR of 1.12 (95% CI 0.88, 1.42). However, notable heterogeneity was observed with an I^2^ of 86% and a *p*-value of 0.009 (Fig. 4A). Two studies [14, 19] were included for the analysis of the relationship between T2 and MACE events, revealing a HR of 1.36 (95% CI 1.12, 1.64) with minimal heterogeneity (Fig. 4B). Similarly, two studies [18, 19] were incorporated to analyze the association between LGE and MACE, showing a HR of 1.10 (95% CI 0.63, 1.90) with minimal heterogeneity (Fig. 4C).

Due to the limited number of literature sources, we were unable to conduct subgroup analyses. We hypothesize that variations in data measurement locations, CMR duration, and hormone administration may all contribute to the emergence of heterogeneity.

### 3.4 Publication Bias

Due to the limited sample size (<10) in our study, the assessment of publication bias is deemed unreliable; consequently, we refrained from conducting such an evaluation [29].

## 4. Discussion

**Fig. 5.**
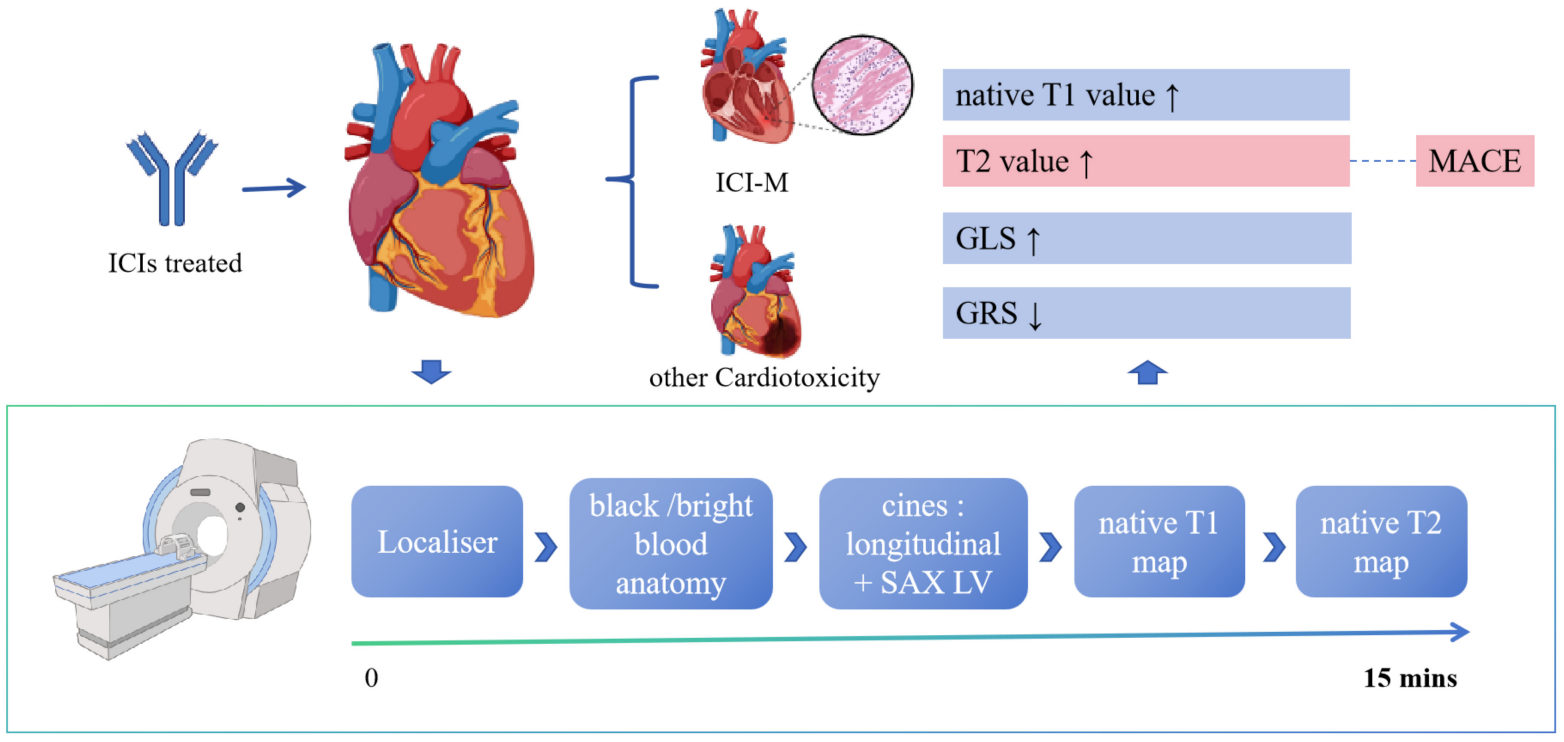
**Summary of the main results**. Patients may develop ICI-M or other cardiotoxicity after using ICIs. At this point, the CMR protocol shown in the figure can be used, with rapid imaging within 15 minutes. The figure details the effects of ICI on cardiac parameters, including increases in T1 and T2 values, increases in GLS, and decreases in GRS. SAX LV, short-axis left ventricle; ICIs, immune checkpoint inhibitors; ICI-M, immune checkpoint inhibitor-associated myocarditis; MACE, major adverse cardiac events; GLS, global longitudinal strain; GRS, global radial strain; CMR, cardiac magnetic resonance. Created with BioRender.com.

This study suggests that following the administration of ICI, patients exhibit changes in parameters reflecting myocardial strain, such as the rise of GLS and the reduction of GRS. Additionally, parameters indicating myocardial tissue characteristics, native T1 and T2, demonstrate an increase, while changes in GLS, ECV, and LGE presence remain inconclusive. Furthermore, this study indicates the occurrence of MACE in patients more likely to incorporate the elevation of T2. (Fig. 5) These significant metrics can all be obtained using a streamlined abbreviated (non-contrast) protocol (Fig. 5), which requires only 15 minutes [30]. This provides the possibility for wider application of CMR in the field of cardio-oncology.

This streamlined abbreviated (non-contrast) protocol included cine balanced steady-state free precession (SSFP) imaging for longitudinal and transverse sections, and native T1 & T2 maps performed in short-axis slices [30]. These data are sufficient for obtaining left ventricular ejection fraction (LVEF), GLS, GRS, GCS, native T1, and native T2. The new CMR protocol reduces the duration of CMR procedures (in which the entire process takes approximately 15 minutes) and concurrently lowers the associated costs (Fig. 5).

### 4.1 CMR-FT can Reflect Subclinical Cardiac Dysfunction Following ICI Usage, which is also Associated with Poor Prognosis

This meta-analysis revealed differences in CMR-FT between patients using ICI or those with pre-existing complications like myocarditis compared to normal populations or reference values. The GLS showed an increase, while the GRS demonstrated a decrease; however, the differences in GCS lacked statistical significance. GLS is recognized as an indicator reflecting endocardial myocardial damage and has been incorporated into 2022 ESC Guidelines for diagnosing CTRCD [9]. Our study reinforces the understanding of how GLS can be utilized in assessing CTRCD during the use of ICI. Meanwhile, GRS has also exhibited notable differences, which were previously overlooked in past studies and may have underestimated its value. In the study by Michel *et al*. [31] on anti-PD-1 therapy, overall longitudinal strain did not exhibit a significant decrease. However, there was a 51% reduction in overall radial strain, indicating a compromised overall left ventricular contractility in mice [32]. GRS has also been mentioned in early monitoring within the mouse model of programmed cell death protein 1 (PD-1) antibody [33]. Changes in myocardial strain may be associated with ICI-induced increased T-cell infiltration in the myocardium and its impact on cardiac energy metabolism [31].

Myocardial strain is correlated with MACE. Ventricular ejection fraction is associated with myocardial wall thickness and strain, with strain serving as an indicator of changes in ventricular ejection fraction [34]. Michel *et al*. [31] discovered that follow-up GLS had a good correlation with the incidence of extra-cardiac irAEs (r = 0.43; *p* = 0.03). Quinaglia *et al*. [35] posit that GLS, GCS, and GRS demonstrate a higher accuracy in predicting MACE compared to LVEF, cardiac troponin T (cTnT), and age post-diagnosis of ICI-M.

In various studies, there is a varied reduction in the absolute values of GLS and GRS. Zhao and others indicated a correlation between patients with GLS >–14.2% and higher MACE risk [15]. Additionally, the ESC guidelines consider a GLS decline of 15% as a diagnostic criterion for CTRCD [9].

### 4.2 The Histological Parameters of CMR can Diagnose ICI-M and also Indicate Prognosis

In this study, patients showed prolonged native T1 and T2 values after receiving ICI, especially in cases of cardiac complications such as myocarditis, while the difference in ECV and LGE presence were not statistically significant. The emergence of myocardial histological parameters in CMR has transformed the previous scenario where myocardial changes relied solely on myocardial biopsy, now offering non-invasive access to myocardial histology. The CMR tissue characterization, such as T1 and T2 values, indeed holds diagnostic value for identifying ICI-related cardiotoxicity. This aligns with the research findings of Altaha in chemotherapy for tumors [36].

The utilization of T1 and T2 values has been incorporated into the 2018 expert recommendations for diagnosing myocarditis [37]. The findings of this study demonstrated that the increased native T1 and T2 values are similarly applicable in myocarditis induced by ICI. However, ICI-M differs to some extent from the classic phenotype of myocarditis. The myocardial edema in ICI-M, even if not entirely absent, tends to be subtle, especially during ongoing corticosteroid treatment [38]. As mentioned by Thavendiranathan *et al*.’s study [14], non-ICI-M often exhibits a more pronounced increase in T2 values. This might reflect variances in the mechanisms and extent of myocardial damage or differences in steroid use among our patients before CMR [14]. The myocardial lymphocytic infiltration has been referenced in both human and animal models of ICI-M [20, 22]. The consistent finding noted on histology was patchy to gross, T-cell-predominant lymphocytic infiltrate within the myocardium, which was similar in findings to those seen in cardiac transplant rejection; no granulomas or giant cells were noted [39].

The outcomes of this study indicated a correlation between the T2 values of oncology patients treated with ICI and MACE events, with a HR of 1.36 (95% CI 1.12, 1.64). However, there was no correlation between T1 values, LGE values, and MACE. This lack of correlation may be attributed to the current scarcity of relevant research. It is important to note that we cannot definitively conclude that T1 and LGE are unrelated to MACE. The study on doxorubicin-induced cardiac toxicity in animals demonstrated that T2 mapping identifies myocardial edema in the reversible stage of cardiac toxicity, whereas T1 mapping and ECV primarily indicate late-stage cardiac toxicity associated with myocardial fibrosis [40]. In some studies, there is a correlation between T1 value and MACE events, which is inconsistent with the findings of this study [41]. This could potentially be associated with the timing of CMR acquisition and the administration of hormones.

The study by Chaikriangkrai *et al*. [42] in heart transplantation showed that higher myocardial T2 was associated with MACE. Conversely, no significant correlation was found between T1 mapping biomarkers and MACE. Chaikriangkrai *et al*. [42] suggested that elevated T2 values are linked to recurrent inflammatory events. Prolonged myocardial edema and inflammation may lead to decreased ventricular compliance and increased stiffness, ultimately resulting in MACE [42]. Our analysis results suggest that ICI-M could entail similar pathophysiological changes post-heart transplantation.

In non-ICI myocarditis, LGE is considered the strongest independent prognostic predictor, with a HR of 8.4 for mortality and a HR of 12.8 for cardiac mortality, significantly associated with non-ICI myocarditis-related MACE [41]. However, our research indicated a weak correlation between ICI-M and LGE-associated MACE events, with a HR of 1.10 (95% CI 0.63, 1.90). This might be attributed to incongruities between the pathophysiological mechanisms of ICI-M and myocardial fibrosis or scarring reflected by LGE [14]. LGE may be a late pathological change of myocardial inflammation and injury, further compounded by the infrequency of LGE in ICI-M [19]. We observed that the differences in ECV among patients using ICI also appear to lack significance, with an SMD of 0.28 (95% CI –0.06, 0.62, *p* = 0.11). This may align with the mechanisms of LGE in patients using ICI.

### 4.3 CMR has Greater Utility and Value in Oncocardiology

In our analysis, we observed differences in GRS and GLS in patients before and after the use of ICI or the occurrence of complications. Perhaps in the future, combining GRS with GLS to assess patients’ cardiac function could potentially further elucidate risk stratification of cardiovascular complications in patients. Subsequently, myocardial work indices (MWIs) could also be employed in conjunction with CMR-FT parameters for cardiac function analysis [43]. Moreover, our research findings indicated that the manifestations of ICI-M resemble myocarditis, yet its pathophysiological mechanisms might differ from those of myocarditis. Subsequent studies on ICI-M could focus not only on myocarditis itself but also on immune-related cardiac toxicity, which may be similar to transplant-related cardiac toxicity. CMR tissue characterization can identify ICI-M as well as myocardial changes post-ICI use. Our data suggests a correlation between patients’ T2 values and MACE, whereas there is a lack of association regarding T1 and LGE. Presently, there remains a scarcity of research concerning the prognostic relevance of CMR tissue characterization in relation to MACE. More clinical research data is needed to emerge in this area. Subsequently, the utilization of ultrasmall superparamagnetic iron oxide (USPIO) for T2* enhancement to detect inflammatory macrophages within the myocardium could also be explored in later stages [44].

### 4.4 Limitations

Due to the recent emergence of ICIs as novel anti-cancer drugs, there has been a limited amount of clinical research on CMR’s response to ICI-induced cardiotoxicity. The article by Thavendiranathan *et al*. [14] did not include baseline data on a healthy control group or patients’ before being treated with ICI, but solely offered local reference values. As this article represents a multi-center large-scale study that we are unwilling to discard, local normal reference values were utilized as the control group, matching the sample size of the experimental group. Acknowledging the potential for error with this approach, we conducted a meta-analysis excluding this article in the **Supplementary Materials** (**Supplementary Figs. 1,2**), revealing no disparities in conclusions. This article aims to include articles with available targeted data to the fullest extent possible. The heterogeneity among studies arises from variations in research designs, differences in the focal points of CMR, and discrepancies in the collected data at different time points, leading to diversity among the articles. Future studies could validate our findings through larger-scale, multicenter research endeavors and delve deeper into exploring the mechanisms underlying the cardiotoxicity of ICIs.

Additionally, due to the small sample size, while acknowledging the diagnostic and prognostic value of CMR in ICI-related cardiotoxicity, the relationship between specific parameters and the outcomes of interest still requires more research. There is a notable scarcity of studies examining the correlation between CMR indicators and MACE. As a result, the conclusions derived from this aspect of the analysis should be approached with caution. We anticipate further research in this area in the future. Moreover, there is currently a lack of data on the optimal frequency of CMR follow-ups for patients using ICIs, and the ambiguity in defining cutoff values for CMR in ICI-related cardiotoxicity limits its application in oncology patients.

## 5. Conclusions

The cardiotoxicity of the novel anti-tumor drug type, ICI, cannot be overlooked. The meta-analysis highlights CMR as a promising diagnostic biomarker for cardiotoxicity, particularly in cases of ICI-related myocarditis and non-inflammatory forms of heart failure associated with ICI therapy. In our investigation, when patients manifested ICI-related cardiotoxicity after a period of ICI use (2–3 months), T1 and T2 exhibited an increase, while GLS increased and GRS decreased. In terms of prognosis, the meta-analysis underscores CMR as a promising indicator of cardiotoxicity associated with ICI therapy. Elevated T2 in ICI-associated myocarditis patients correlates with an elevated risk of MACE. We summarized various indicators and identified a streamlined abbreviated (non-contrast) protocol that includes significant indicators, allowing for CMR scanning to be completed within 15 minutes, which is advantageous for the promotion of CMR in cardio-oncology. Expanding the applications of CMR for the non-invasive detection of ICI-related cardiotoxicity could enhance clinical diagnosis and treatment, thereby improving the monitoring and management of patients undergoing ICI therapy.

## Availability of Data and Materials

All data points generated or analyzed during this study are included in this article and there are no further underlying data necessary to reproduce the results.

## Supplementary Material

Supplementary material associated with this article can be found, in the online version, at https://doi.org/10.31083/RCM25508.
