# Supplementary material for: Diagnostic and Prognostic Value of Cardiac Magnetic Resonance for Cardiotoxicity Caused by Immune Checkpoint Inhibitors: A Systematic Review and Meta-Analysis
Source: Rev Cardiovasc Med. 2025 Feb 21;26(2):25508. doi: 10.31083/RCM25508 (PMC11868891; doi:10.31083/RCM25508)
Supplement: Supplementary file 1 [file 2153-8174-26-2-25508-s1.zip › Supplementary materials.docx]

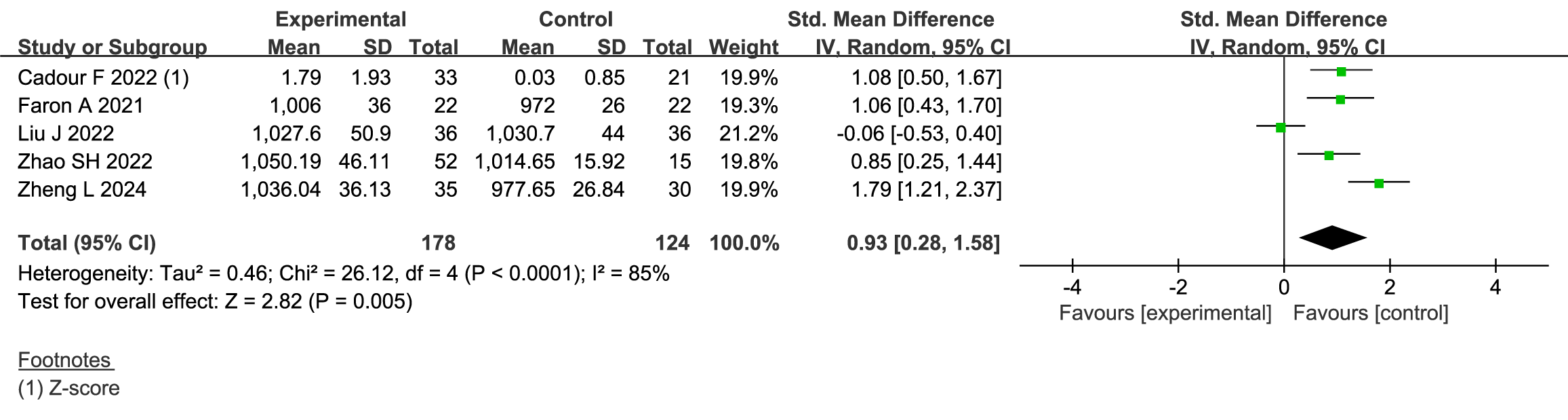


**Figs.1 T1 of ICI-related cardiac toxicity.** Z-score provides an assessment of how many SDs each patient's T1 value deviates from the mean within the normal range for each site, vendor, and CMR field strength. CMR, cardiac magnetic resonance; ICI, immune checkpoint inhibitors; CI, confidence interval.


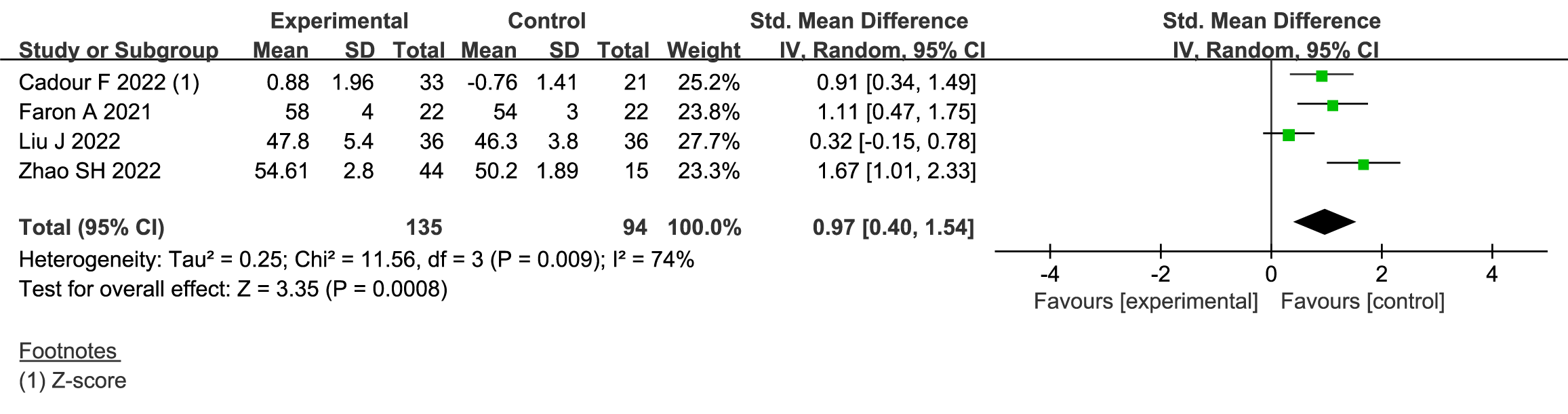


**Figs. 2 T2 of ICI-related cardiac toxicity.** Z-score provides an assessment of how many SDs each patient's T2 value deviates from the mean within the normal range for each site, vendor, and CMR field strength. CMR, cardiac magnetic resonance; ICI, immune checkpoint inhibitors; CI, confidence interval.
